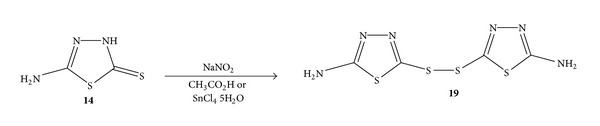# Synthesis and *In Vitro* Evaluation of Novel Acyclic and Cyclic Nucleoside Analogs with a Thiadiazole Ring

**DOI:** 10.1155/2013/159164

**Published:** 2013-03-05

**Authors:** Yuxiang Zhao, Peter J. McCarthy, Cyril Párkányi

**Affiliations:** ^1^Division of Science and Mathematics, Eureka College, 300 E. College Avenue, Eureka, IL 61530, USA; ^2^Division of Biomedical Marine Research, Harbor Branch Oceanographic Institute, Florida Atlantic University, Fort Pierce, FL 34946, USA; ^3^Department of Chemistry and Biochemistry, Florida Atlantic University, 777 Glades Road, P.O. Box 3091, Boca Raton, FL 33431-0991, USA

## Abstract

The synthesis of six thiadiazole nucleoside analogs is reported: 5-diacetylamino-1,2,4-thiadiazol-3-one (**1**), 5-amino-2- (tetrahydrofuran-2-yl)-1,2,4-thiadiazol-3-one (**2**), 5-amino-3-[(2′-hydroxyethoxy)methyl]-1,3,4-thiadiazol-2-one (**3**), 5-amino-3-(4′-hydroxy-2′-hydroxymethyl-butyl)-1,3,4-thiadiazole-2-thione (**4**), (R)-5-amino-3-(2′,3′-dihydroxypropyl)-1,3,4-thiadiazole-2-thione (**5**), and (S)-5-amino-3-(2′,3′-dihydroxypropyl)-1,3,4-thiadiazole-2-thione (**6**). The synthesis, characterization, and properties of these new synthesized thiadiazole derivatives are discussed. A dimerization of 5-amino-3H-1,3,4-thiadiazole-2-thione (**14**) by sodium nitrite resulting in di-(5-amino-1,3,4-thiadiazol-2-yl) disulfide (**19**) is also reported. The preliminary *in vitro* evaluation of these newly synthesized compounds is discussed.

## 1. Introduction

Since Heidelberger et al. discovered 5-fluorouracil, a pyrimidine antimetabolite, in 1957, pyrimidine antimetabolites have become one of the most important groups of anticancer agents. Some pyrimidine nucleosides have been proven to possess promising anticancer activity [1–8], such as Floxuridine, Cytarabine, and Gemcitabine. Many analogs of pyrimidine nucleosides are active against viruses as well. For example, Zidovudine (Azidothymidine, AZT or ZDV), Lamivudine, Idoxuridine, Trifluridine, and Cytarabine are all used for treating viral infections [9–23]. A number of these highly successful antiviral compounds are due to the collaboration between Dracinsky et al. [17] and de Clercq and Holý [17–21] (Viread, Truvada, Atripla, Lamivudine, Vistide, Hepsera). In our laboratory, we have focused on the development of novel antimetabolites for many years, including some compounds with a thiadiazole ring. Experimental evidence indicates similarities in physical and chemical properties between a –CH=CH– bond in aromatic hydrocarbons and bivalent sulfur, –S–, in sulfur heterocycles [24, 25]. For this reason, 5-amino-2H-1,2,4-thiadiazol-3-one and 5-amino-3H-1,3,4-thiadiazol-2-one can be considered as the analogs of cytosine. Based on this analogy, within the framework of our systematic studies, we have synthesized some novel acyclic or cyclic nucleoside analogs with a thiadiazole ring instead of a pyrimidine ring.

## 2. Results and Discussion

**Figure 1:**
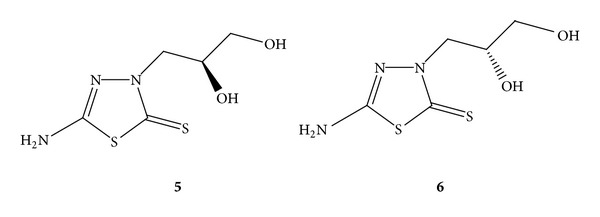


In the present paper, we report the preparation of 5-diacetylamino-1,2,4-thioadiazol-3-one (**1**) and five thiadiazole-based nucleoside analogs: 5-diacetylamino-1,2,4-thiadiazol-3-one (**1**), 5-amino-2 (tetrahydrofuran-2-yl)-1,2,4-thiadiazol-3-one (**2**), 5-amino-3-[(2′-hydroxyethoxy)-methyl]-1,3,4-thiadiazol-2-one (5-amino-3-(4′-hydroxy-2′-hydroxymethyl-butyl)-1,3,4-thiadiazole-2-thione (**4**), (S)-5-amino-3-(2′,3′-dihydroxypropyl)-1,3,4-thiadiazole-2-thione (**5**), and (R)-5-amino-3-(2′,3′ dihydroxypropyl)-1,3,4-thiadiazole-2-thione (**6**). **5 **and **6 **are stereoisomers (see Figure 1). Their racemic mixture **7** was also prepared and tested.

### 2.1. Preparation of 5-Diacetylamino-1,2,4-thiadiazol-3-one **(1)** and 5-amino-2-(tetrahydrofuran-2-yl)-1,2,4-thiadiazol-3-one **(2)**

**Scheme 1:**
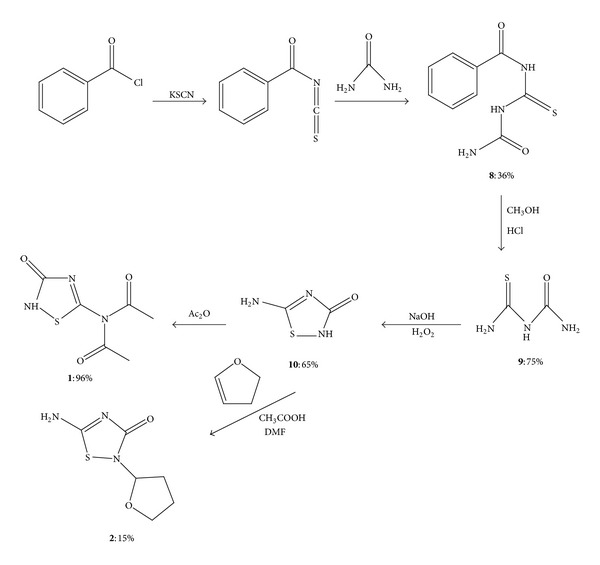


The synthesis of 5-diacetylamino-1,2,4-thiadiazol-3-one (**1**) and 5-amino-2-(tetrahydrofuran-2-yl)-1,2,4-thiadiazol-3-one (**2**) is shown in Scheme 1. 5-Amino-2H- 1,2,4-thiadiazol-3-one (**10**) was prepared first according to a known method, and then based on **10,** 1,2,4-thiadiazole derivatives **1 **and **2 **were produced. The preparation of **10** was carried out using modified procedures based on an approach by Kurzer [26] and Kurzer and Taylor [27]. The starting materials were benzoyl chloride and potassium thiocyanate. Potassium thiocyanate (KSCN) reacted with benzoyl chloride by the acylation reaction. Benzoylisothiocyanate reacted *via *addition reaction with urea to give **8.** The yield for the first two steps of this synthetic method was 36%. N-Ureidocarbothioyl benzamide **(8) **was debenzoylated by refluxing with methanol and hydrochloric acid. Thiobiuret (**9**) was obtained as light yellow crystals in a 75% yield. In the next step, with the addition of NaOH and H_2_O_2_ at 0–5°C, a condensation-cyclization reaction was carried out and resulted in 5-amino-2H-1,2,4-thiadiazol-3-one (**10**) as white crystals in a 65% yield. The structures of the known compounds were determined on the basis of their analytical and spectral data, such as IR, ^1^H NMR, and ^13^C NMR, and melting point. These data were in agreement with not only their proposed structures but also the information reported in the literature.

Compound **10** reacted with acetic anhydride at room temperature to produce 5-diacetylamino-2H-1,2,4-thiadiazol-3-one (**1**), with a 96% yield. The structure of 5-diacetylamino-2H-1,2,4-thiadiazol-3-one (**1**) was determined on the basis of its analytical and spectral data.

In the presence of acetic acid, **10** was refluxed with two equivalents of 2,3-dihydrofuran to afford 5-amino-2-(tetrahydrofuran-2-yl)-1,2,4-thiadiazol-3-one (**2**) as a white solid in 15% yield. This method was modified based on Lukevics' procedures. He studied the reaction between 2,3-dihydrofuran and 5-fluorouracil under various conditions [28]. In our approach, we avoid using high pressure or any strong acids, since thiadiazole ring may be cleaved by acids. 2,3-Dihydrofuran was protonated by acetic acid. Electrophilic attack on annular nitrogen by protonated 2,3-dihydrofuran gave **2 **as the product. The annular nitrogen in 1,2,4-thiadiazole is reactive towards electrophiles. 

The structure of 5-amino-2-(tetrahydrofuran-2-yl)-1,2,4-thiadiazol-3-one (**2**) was determined by its analytical and spectral data which are in agreement with the proposed structure [2]. 

### 2.2. Preparation of 5-Amino-3-[(2′-hydroxyethoxy)methyl]-1,3,4-thiadiazol-2-one **(3)**

**Scheme 2:**
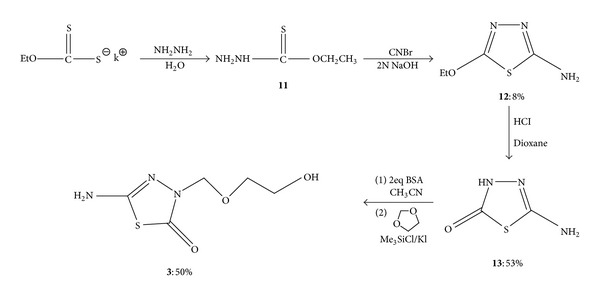


The synthesis of 5-amino-3-[(2′-hydroxyethoxy)methyl]-1,3,4-thiadiazol-2-one (**3**) is shown in Scheme 2. 5-Amino-3H-1,3,4-thiadiazol-2-one (**13**) was prepared first, and then, based on **13**, 1,3,4-thiadiazole derivative **3** was obtained. The synthesis of **13** was not initially successful, with the synthetic approach based on Freund and Schander's procedure [29]. They claimed that 5-amino-3H-1,3,4-thiadiazol-2-one **(13)** was prepared by heating 2-thiodiurea with concentrated hydrochloric acid.

The literature shows two other possible synthetic approaches. Rüfenacht [30] used potassium methyl xanthate and hydrazine hydrate to form *O*-methylester. Phosgene was introduced to *O-*methyl ester to produce 5-amino-2-methoxy-1,3,4-thiadiazole by a cyclization reaction. Subsequently, the hydrolysis of 5-amino-2-methoxy-1,3,4-thiadiazole yielded **13 **as the product. Clarkson and Landquist [31] reported a similar approach, but they used cyanogen chloride for the cyclization reaction. Considering that both phosgene and cyanogen chloride are war gases and not easy to handle, solid cyanogen bromide was used in our synthesis to perform this cyclization reaction. Following Clarkson and Landquist's procedure, 2-amino-5-ethoxy-1,3,4-thiadiazole **(12) **was successfully synthesized in an 80% yield. The hydrolysis of **12 **with a trace of hydrochloric acid gave **13 **as white crystals in 53% yield. The structures of compounds **11–13** were identified by their analytical and spectral data. These data were also compared with literature values for identification.

After the preparation of 5-amino-3H-1,3,4-thiadiazol-2-one (**13**), acyclonucleoside analog **3 **was successfully synthesized by a convenient one-pot method. This synthetic method was based on modified reactions reported by Keyser et al. [32] and Ubasawa et al. [33]. Keyser et al. reported that 1,3-dioxolane can react with iodotrimethylsilane at −78°C under nitrogen to produce iodomethyl [(trimethylsilyl)oxy]ethyl ether, which could react with purine or pyrimidine sodium salt to form acyclonucleosides. Ubasawa et al. reported a similar reaction with silylated pyrimidine bases instead of the sodium salt. Our synthetic approach used silylated 1,3,4-thiadiazole to perform this reaction. Bis(trimethylsilyl) acetamide was stirred with **13 **to produce bis-trimethylsilyl-1,3,4- thiadiazole, which was allowed to react with 1,3-dioxolane, chlorotrimethylsilane, and potassium iodide without further separation to yield 5-amino-3-[(2′- hydroxyethoxy)methyl]-1,3,4-thiadiazol-2-one (**3**).

The structure of 5-amino-3-[(2′-hydroxyethoxy)methyl]-1,3,4-thiadiazol-2-one (**3**) was determined on the basis of its analytical and spectral data, which are in agreement with the proposed structure.

### 2.3. Preparation of 5-Amino-3-(4′-hydroxy-2′-hydroxymethyl-butyl)-1,3,4-thiadiazole-2-thione **(4)**

**Scheme 3:**
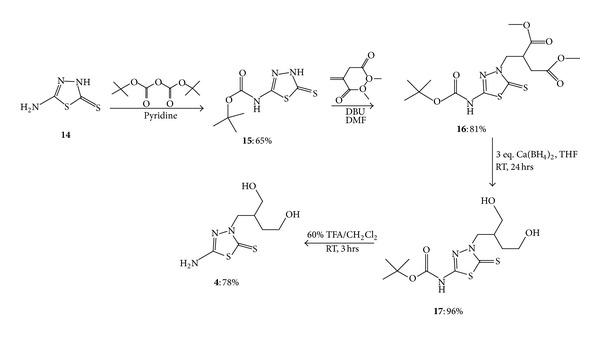


In the course of the preparation of nucleoside analogs, purine and pyrimidine bases are generally introduced by substitution. Only a few acyclic nucleosides were synthesized by Michael addition [34–37]. The Michael-type addition uses amino groups as the nucleophiles and not enolate anions as in the original Michael addition. It is interesting to prove that this strategy could work with thiadiazoles as well. On the basis of previous publications, a synthetic method was modified to make the reactions with 5-amino-3H-1,3,4- thiadiazole-2-thione (**14**) plausible (Scheme 3).

5-Amino-3H-1,3,4-thiadiazole-2-thione (**14**) contains a primary amino group, which needs to be protected in this synthetic approach. A very common protecting group, Boc (tert-butoxycarbonyl), was introduced, and it worked with this synthetic method very well. 5-Amino-3H-1,3,4-thiadiazole-2-thione (**14**) was treated with di-tert-butyl dicarbonate (Boc anhydride) to produce 5-tert-butylcarbamate-3H-1,3,4-thiadiazol-2-thione (**15**) as a light yellow solid in 65% yield. Nucleophile **15 **reacted with Michael acceptor dimethyl itaconate at room temperature to afford dimethyl 2-[(5-(tert-butoxycarbonyl)-2-thioxo-1,3,4-thiadiazol-3-yl)methyl] succinate (**16**) in 81% yield. A strong base, 1,8-diazabicyclo[5.4.0]undec-7-ene (DBU), was used in this Michael-type addition reaction. In the next step, two ester groups in **16 **were reduced to two alcohol groups by Ca(BH_4_)_2_. Ca(BH_4_)_2_ was prepared *in situ *by treating NaBH_4_ with calcium chloride. A clear gel-like product obtained after this reduction reaction was 5-tert- butoxycarbonyl-3-[4′-hydroxy-2′-(hydroxymethyl)butyl]-1,3,4-thiadiazole-2-thione (**17**). Although attempts to produce 5-tert-butoxycarbonyl-3-[4′-hydroxy-2′-(hydroxymethyl)butyl]-1,3,4-thiadiazole-2-thione (**17**) with other reducing agents, such as NaBH_4_, DIBAL (diisobutylaluminum hydride), or LiAlH_4_, were also carried out, Ca(BH_4_)_2_ was proven to afford the highest yield, 96%. It appears that DIBAL and LiAlH_4_can cleave the thiadiazole ring. A Boc deprotection of compound **17 **by 60% trifluoroacetic acid (TFA) in methylene chloride at room temperature reproduced the primary amino group and afforded the final product **4 **in 78% yield.

The analytical and spectral data of 5-tert-butylcarbamate-3H-1,3,4-thiadiazole-2-thione (**15**), dimethyl2-[(5-(tert-butoxycarbonyl)-2-thioxo-1,3,4-thiadiazole[-3-yl)methyl]succinate **(16)**, 5-tert-butoxycarbonyl-3-[4′-hydroxy-2′-(hydroxymethyl)butyl]-1,3,4-thiadiazole-2-thione **(17),** and 5-amino-3-(4′-hydroxy-2′-hydroxymethyl-butyl)- 1,3,4-thiadiazole-2-thione (**4**) are in agreement with the proposed structure.

### 2.4. Preparation of 5-Amino-3-(2′,3′-dihydroxypropyl)-1,3,4-thiadiazole-2-thione

**Scheme 4:**
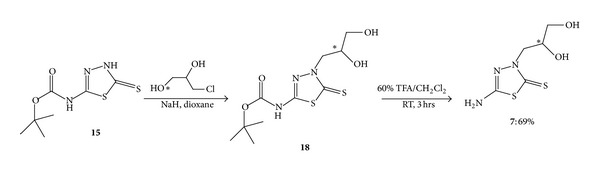


The racemic mixture of (S)-5-amino-3-(2′,3′-dihydroxypropyl)-1,3,4-thiadiazole-2- thione (**5**) and (R)-5-amino-3-(2′,3′-dihydroxypropyl)-1,3,4-thiadiazole-2-thione (**6**) was prepared by using the synthetic method described in Scheme 4. The protection of the primary amino group in 5-amino-3H-1,3,4-thiadiazole-2-thione (**14**) using Boc anhydride was the same as the previous reactions in Scheme 3. Then, **15 **was converted into a sodium salt with sodium hydride in anhydrous dioxane, followed by a nucleophilic substitution reaction with racemic 3-chloro-1,2-propanediol to obtain 5-tert-butoxycarbonyl-3-(2′,3′-dihydroxypropyl)-1,3,4-thiadiazole-2-thione (**18**). The experimental procedure for this nucleophilic substitution reaction was modified on the basis of a similar reaction reported by Párkányi et al. [38] in 1989. The deprotection of the crude product **18 **with 60% trifluoroacetic acid in methylene chloride restored the primary amine. This reaction removed the Boc group and obtained acyclic nucleoside analog **7** as a racemic mixture of **5 **and **6 **in a 69% yield. To prepare a pure enantiomer with the same approach, (R)-3-chloro-1,2-propanediol was used to yield (S)-5-amino-3-(2′,3′-dihydroxypropyl)-1,3,4-thiadiazole-2-thione **(5)** and the (S)-3-chloro-1,2-propanediol was used to yield (R)-5-amino-3-(2′,3′-dihydroxypropyl)-1,3,4-thiadiazole-2-thione (**6**).

As enantiomers, **5 **and **6 **have identical melting points, boiling points, infrared spectra, NMR spectra, and solubility. Because the most common property in which enantiomers differ is their behavior toward plane-polarized light, specific rotation was measured to identify the separate enantiomers **5 **and **6**. Specific rotation of the (S)-5-amino-3-(2′,3′- dihydroxypropyl)-1,3,4-thiadiazole-2-thione (**5**) was [*α*]_D20_ = −33.0° (*c* = 1.0, CH_3_OH), and specific rotation of the (R)-5-amino-3-(2′,3′-dihydroxypropyl)-1,3,4-thiadiazole-2-thione (**6**) was [*α*]_D20_ = +31.1° (*c* = 0.9, CH_3_OH). These results proved that separate enantiomers **5 **and **6 **have been obtained. 

### 2.5. Preparation of Bis-(5-amino-1,3,4-thiadiazol-2-yl) Disulfide **(19)** by Dimerization

In our attempts to carry out a diazotization reaction, we discovered an interesting dimerization reaction. The reaction was designed to use sodium nitrite and an acid to prepare nitrous acid *in situ, *which could react with **14 **to form a diazonium salt. Then, this diazonium group could be replaced by a functional group such as OH. Various experimental conditions and acids have been used to study this reaction. After treating **14** with sodium nitrite and acetic acid in an ice bath, a yellow solid was produced in 73% yield. Surprisingly, the NMR and mass spectrometry have shown that this yellow product was not the compound we proposed from a diazotization reaction, but a disulfide, bis-(5- amino-1,3,4-thiadiazol-2-yl) disulfide **(19)** (Scheme 5). The primary amino group on the aromatic thiadiazole ring was not affected by the diazotization reaction at all. Besides acetic acid, another reagent-stannic chloride (SnCl_4_·5H_2_O) was also used with sodium nitrite to yield **19 **successfully in 68% yield.

It is of interest to report that sodium nitrite with an acid or stannic chloride can oxidize **14 **to produce disulfide **19. **The preparation of **19 **was reported by Mahieu et al. in 1986 with sulfonyl chloride as the oxidant [39]. Another oxidizing agent reported for this reaction was sodium chlorite, which was described by Ramadas and Srinivasan [40] and Ramadas et al. [41]. Our new reaction conditions can represent a good addition to the synthesis of disulfide **19. **The structure of **19 **was determined by IR, ^1^H NMR,^13^C NMR, and the melting point. These data were in agreement with not only the proposed disulfide structure but also the literature values [39, 40] for identification and confirmation. 

## 3. Bioactivity Results and Discussion

All new synthesized thiadiazole acyclic and cyclic nucleoside analogs (**1–7**) and disulfide **19** were tested for cytotoxicity against P388 murine leukemia cells, PANC-1 human pancreatic cancer cells, A549 nonsmall cell lung adenocarcinoma cells, DLD-1 human colon cancer cells, and NCI/ADR drug-resistant human breast cancer cells. These structures of thiadiazole nucleoside analogs did not prove to be significantly cytotoxic to the tumor tissue cultures at concentrations of 5 *μ*g/mL. This result did not surprise us because most of our target compounds are acyclic nucleosides, which are mostly antiviral agents and not anticancer agents. 

Compounds **1–7** and **19 **were also tested for their antimicrobial activity against *Candida albicans*, *Staphylococcus aureus*, *Pseudomonas aeruginosa*, and methicillin-resistant *Staphylococcus* aureus (MRSA). Better results were obtained in screening for antimicrobial activity. Two compounds, **3 **and **19**, were active against *Staphylococcus aureus* and MRSA at 50 *μ*g per disc. The minimum inhibitory concentration (MIC) for compound **3 **toward S. *aureus* was >50 *μ*g/mL, and toward MRSA was >50 *μ*g/mL as well. MIC of disulfide **19 **toward *S*. aureus was 50 *μ*g/mL and toward MRSA was 25 *μ*g/mL. 

In summary, the thiadiazole acyclic and cyclic nucleoside analogs **1–7** and disulfide **19 **are not active against five available kinds of cancer cells. Although they are not cytotoxic, it was noted that **3 **and **19** possess considerable antibacterial activity against *S*. *aureus* and MRSA. 

## 4. Experimental

The melting points were determined on a Fisher-Johns melting point apparatus (W.H. Curtin & Co.) or Mel-Temp (Electrothermal). ^1^H and ^13^C NMR spectra were recorded with a Varian 400-MHz spectrometer. Infrared spectra were measured on a 4020 GALAXY series FT-IR spectrometer (Mattson Instruments) (potassium bromide disk), or on an Avatar 320 FT-IR spectrometer (Nicolet Instruments). UV spectra were measured on a Cary 3 UV-visible spectrophotometer. Thin layer chromatography (TLC) used silica gel 60 F-254 precoated plates, and the spots were located in the UV light or by iodine vapor. Low resolution MS spectra were recorded on an M-8000 Hitachi mass spectrometer with an L-7100 pump and ion trap mass analyzer. All low resolution mass spectra were obtained in ESI positive mode. High resolution mass spectra were determined by Mass Spectrometry Services at University of Florida, Gainesville, FL, USA. Elemental analyses were performed by Desert Analytics, Tucson, AZ, USA. Specific rotation measurements were conducted at Perkin-Elmer model 141 polarimeter by Dr. David A. Lightner at the University of Nevada, Reno, NV, USA. All solvents used were reagent grade, except for dimethyl sulfoxide, chloroform, acetone, and methanol used in NMR spectroscopic measurements. 

*N-Ureidocarbothioyl Benzamide* (**8**) [41]. Potassium thiocyanate was predried with anhydrous tetrahydrofuran (THF) by stirring overnight. Then, the white powder was filtered off and dried under vacuum on the rotary evaporator to remove THF. A solution was prepared by the addition of 48 g (0.49 mol) potassium thiocyanate in 600 mL toluene. To this solution, 60 mL (0.50 mol) benzoyl chloride was added dropwise with stirring. The solution became milky white after the addition of benzoyl chloride. The mixture was refluxed for 4 hours under argon. The color changed from white to orange. Then, the solution was cooled to room temperature, the white precipitate was filtered off, and the amber filtrate was refluxed with 24.0 g urea (0.40 mol) for 5 hours. Then the reaction mixture was cooled to room temperature and placed in an ice bath for 2 hours to form the crystals. The solution was stirred periodically, and the walls of the flask were scratched when the solution was in the ice bath. After crystallization, bright yellow crystals (33.39 g) were filtered off from the cold solution and dried. This was the crude product, mp 168–171°C. Recrystallization from acetonitrile yielded 32.06 g of bright yellow solid, yield 36%, mp 174-175°C (lit. mp: 175°C [38]). ^1^H NMR (DMSO-d_6_): 7.87–7.89 ppm (m, 2CH in benzene), 7.65–7.68 ppm (m, 2CH in benzene), 7.54–7.58 ppm (m, CH in benzene), 5.39 ppm (s, 4H). ^13^C NMR (DMSO-d_6_): 180.01, 161.20, 133.97, 129.64, 128.54 ppm. IR (KBr): 3354 (m, NH_2_), 3207 (m, N–H), 3001 (m, aromatic C–H), 1716 (s, C=O), 1540 (s, NH_2_ bending), 1493 (s, N–H bending) cm^−1^. MS *m/z*: 223.90 (M + H)^+^.

Thiobiuret (**9**). *N*-*ureidocarbothioyl* benzamide (11 g, 49 mmol) was added to a solution of 220 mL methanol with 0.5 mL of concentrated HCl. The mixture was refluxed for 55 hours. The reaction mixture was cooled to room temperature, and then all the solvent was evaporated. The yellow residue was washed with 5 mL of hexane twice to remove the methyl benzoate and then dried under reduced pressure. Recrystallization from water yielded 4.34 g of light yellow powder, yield 75%, mp 176-177°C. The second crystallization from acetonitrile gave a white powder, mp: 186–188°C (lit. mp: 189–193°C [38]). ^1^H NMR (DMSO-d_6_): 9.69 ppm (s, 1H), 9.47 ppm (s, 1H in NH_2_), 8.96 ppm (s, 1H in NH_2_), 6.95 ppm (s, 1H in NH_2_), 6.29 ppm (s, 1H in NH_2_). ^13^C NMR (DMSO-d_6_): 181.69 ppm, 155.15 ppm. IR (KBr): 3439 (m, NH_2_), 3251 (m, N–H), 1717 (s, C=O), 1580 (s, NH_2_ bending), 1490 (s, N–H bending) cm^−1^.

*5-Amino-2H-1,2,4-thiadiazol-3-one* (**10**). Thiobiuret **9 **(5 g, 42 mmol) was dissolved in 32 mL of a 2 M NaOH solution. The cloudy solution was stirred in ice bath for one hour, and then 6 mL 30% H_2_O_2_ was added dropwise. After stirring in the ice bath for 2 hours, this mixture was neutralized with 3 M HCl to pH 6. After neutralization, a white precipitate was formed. The mixture was filtered and 3.48 g of crude product was collected. The crude product was recrystallized from boiling water to give 3.18 g 5-amino-2H-1,2,4-thiadiazol-3-one **(10)** as fine white crystals, yield 65%, mp 218–221°C (lit. mp: 220–222°C [38]). ^1^H NMR (DMSO-d_6_): 9.33 ppm (s, NH), 8.06 ppm (s, NH_2_). ^13^C NMR (DMSO-d_6_): 176.23 ppm, 168.31 ppm. IR (KBr): 3407 (m, NH_2_), 3059 (m, N–H), 1651 (s, C=O), 1557 (s, NH_2_ bending), 1540 (s, NH bending), 1380 (s, aromatic C=N) cm^−1^.

*5-Diacetylamino-1,2,4-thiadiazol-3-one* **(1)**. 5-Amino-2H-1,2,4-thiadiazol-3-one (**10**) (0.117 g, 1 mmol) was added to 2 mL of acetic anhydride solution, and the mixture was stirred for 5 hours. The white solid was collected by filtration. The product was washed with 3 mL cold water twice and dried under reduced pressure. 5-Diacetylamino-1,2,4-thiadiazol-3-one **(1)** 0.19 g was collected, yield 96%, mp 247-248°C. ^1^H NMR (DMSO-d_6_): 13.39 ppm (s, NH), 2.26 ppm (s, 2CH_3_). ^13^C NMR (DMSO-d_6_): 174.78 ppm, 170.73 ppm, 168.98 ppm, 158.07 ppm, 24.13 ppm, 22.31 ppm. IR: 3118 (m, N–H), 2882 (m, CH_3_), 1683 (s, C=O), 1552 (s, C=N), 1368 (s, C–N) cm^−1^. MS *m/z*: 201.9 M^+^. HRMS: Anal. Calcd. for C_6_H_7_N_3_O_3_S: 202.0281. Found: 202.0287.

*5-Amino-2-(tetrahydrofuran-2-yl)-1,2,4-thiadiazol-3-one* (**2**). 5-Amino-2H-1,2,4-thiadiazole-3-one (**10**) 1.117 g (9.5 mmol) was added to a mixture of 5 mL 2,3-dihydrofuran and 10 drops of glacial acetic acid in 15 mL of DMF. Then, it was refluxed for 6 hours in an oil bath (after the cloudy mixture became clear, it was refluxed for an additional hour). After the reaction was completed, the solvent was evaporated under vacuum and 5-amino-2-(tetrahydrofuran-2-yl)- 1,2,4-thiadiazol-3-one **(2)** was separated by column chromatography. 0.26 g white crystals were collected, yield 15%, mp 150–152°C. ^1^H NMR (CDCl_3_): 1.71 ppm (m, 1H from CH_2_), 1.89 ppm (m, CH_2_), 2.16 ppm (m, 1H from CH_2_), 3.82 ppm (m, 1H from CH_2_), 3.93 ppm (m, 1H from CH_2_), 5.59 ppm (s, CH on furan), 9.66 ppm (s, NH_2_). ^13^C NMR (acetone-d_6_): 183.38 ppm, 153.93 ppm, 81.65 ppm, 67.15 ppm, 32.02 ppm, 24.79 ppm. IR: 3337 (m, NH_2_), 2990 (m, CH_2_), 2878 (m, C–H), 1677 (s, C=O), 1589 (s, C=N), 1537 (s, NH_2_ bending), 1037 (s, C–O–C) cm^−1^. MS *m/z*: 187.97 (M+H)^+^. HRMS: Anal. Calcd. for C_6_H_9_N_3_O_2_S: 190.0634 (M +3H)^3+^. Found: 190.0641. 

*2-Amino-5-ethoxy-1,3,4-thiadiazole* (**12**). Ethylxanthic acid potassium salt (80 g, 0.46 mol) and 40 mL of water were mixed with vigorous stirring to make a paste. Then 24 g (0.6 mol) of 99% hydrazine monohydrate was added to the paste with vigorous stirring. The mixture was stirred for 4 hours at room temperature and then placed in a separatory funnel. When the two layers separated in the separatory funnel, the bottom layer was removed and the top layer was light yellow-greenish oil, which was collected as the crude product, ethyl thiocarbazate **(11)**. This oil was used as the substrate for the next step without further purification. The crude ethyl thiocarbazate **(11)** 9.6 g was dissolved in 48 mL of 2 M NaOH solution and put in an ice bath for 45 minutes. A solution of 8.2 g cyanogen bromide in 40 mL ethanol was added to the above reaction mixture dropwise with stirring. Upon addition of cyanogen bromide, a light yellow precipitate was formed. This mixture was kept in ice bath with stirring for 45 minutes. The light yellow precipitate was collected by filtration. After drying under reduced pressure, 8.83 g light yellow solid was collected, yield 80%. To obtain the analytical sample the product was recrystallized from ethanol, mp 195–202°C (lit. mp: 190–202°C [31]). ^1^H NMR (DMSO-d_6_): 6.72 ppm (s, NH_2_), 4.29 ppm (q, CH_2_), 1.29 ppm (t, CH_3_). ^13^C NMR (DMSO-d_6_): 165.45 ppm, 162.80 ppm, 68.08 ppm, 15.01 ppm. IR: 3293 (m, NH_2_), 3134 (m, N–H), 2986 (m, CH_3_), 2931 (m, CH_2_), 1605 (m, NH_2_ bending), 1562 (s, C=N), 1497 (s, CH_2_ bending), 1014 (s, C–O–C) cm^−1^. 

*5-Amino-3H-1,3,4-thiadiazolin-3-one* (**13**). 2-Amino-5-ethoxy-1,3,4-thiadiazole (**12**) (5 g, 34.5 mmol) was added to a solution of 50 mL dioxane with 3.3 mL concentrated HCl. The mixture was refluxed for 4.5 hours. Then, the solvent was evaporated under reduced pressure. The light brown solid residue was washed with 3 mL ether three times. After recrystallization from water, 2.16 g off-white crystals were collected, yield 53%, mp 170–172°C (lit. mp: 170–172°C [31]). ^1^H NMR (DMSO-d_6_): 11.26 ppm (s, NH), 6.46 ppm (s, NH_2_). ^13^C NMR (DMSO-d_6_): 169.99 ppm, 153.64 ppm. IR: 3321 (m, NH_2_), 3156 (m, N–H), 1658 (s, C=O), 1638 (m, NH_2_ bending), 1562 (s, C=N), 1353 (s, C–N) cm^−1^. HRMS: Anal. Calcd. for C_2_H_3_N_3_OS: 256.9886 (2 M + Na)^+^. Found: 256.9883.

*5-Amino-3-[(2 *′*-hydroxyethoxy)methyl]-1,3,4-thiadiazol-2-one* (**3**). 5-Amino-3H-1,3,4-thiadiazolin-3-one **(13)** (0.60 g, 0.005 mol) was suspended in 10 mL of anhydrous CH_3_CN. 2.76 mL (0.01 mol) of bis(trimethylsilyl)acetamide was added dropwise. The mixture turned clear after several minutes. To this solution, 0.7 mL (0.01 mol) 1,3-dioxolane, 1.7 g (0.01 mol) KI, and 1.38 mL (0.01 mol) chlorotrimethylsilane were added. The mixture was stirred for 16 hours at room temperature under argon. After the reaction, 20 mL of methanol was added to quench the reaction, and the reaction mixture changed from light yellow to dark brown. NaHCO_3_ was added to neutralize the mixture to pH 8. Then, the solvent was evaporated under reduced pressure, and the residue was loaded onto a silica gel column for purification. The final product was 0.52 g, yield 50%, mp 65–67°C. ^1^H NMR (DMSO-d_6_): 7.21 ppm (s, NH_2_), 5.37 ppm (s, CH_2_), 4.67 ppm (t, OH), 3.60 ppm (t, CH_2_), 3.49 ppm (m, CH_2_). ^13^C NMR (DMSO-d_6_): 181.81 ppm, 157.75 ppm, 77.04 ppm, 71.64 ppm, 59.85 ppm. IR: 3410 (m, NH_2_), 3292 (m, OH), 2925 (m, CH_2_), 1600 (s, C=O), 1568 (m, NH_2_ bending), 1412 (s, C=N), 1350 (s, C–N), 1100 (s, C–O–C) cm^−1^. HRMS: Anal. Calcd. for C_5_H_9_N_3_O_3_S: 214.0257 (M + Na)^+^. Found: 214.0274. 

*5-tert-Butylcarbamate-3H-1,3,4-thiadiazol-2-thione* (**15**). 5-Amino-3H-1,3,4-thiadiazole-2-thione (**14**) [42] (1.06 g, 8 mmol) was dissolved in 20 mL pyridine. 1.74 g (8 mmol) melted (Boc)_2_O was added to the mixture dropwise and stirred at room temperature overnight. Pyridine was evaporated under reduced pressure, and the residue was washed with diethyl ether twice, followed with recrystallization from CH_3_Cl. 1.21 g off-white solid was collected, yield 65%. mp: 157–159°C. ^1^H NMR (DMSO-d_6_): 13.92 ppm (s, NH), 11.87 ppm (s, NH), 1.45 ppm (s, 3CH_3_). ^13^C NMR (DMSO-d_6_): 183.42 ppm, 154.12 ppm, 152.65 ppm, 83.17 ppm, 28.46 ppm. IR: 3145 (m, N–H), 1704 (s, C=O), 1578 (m, NH_2_ bending), 1545 (s, C=N), 1305 (s, C–N), 1071 (s, C–O) cm^−1^. HRMS: Anal. Calcd. for C_7_H_11_N_3_O_2_S_2_: 256.0185 (M+Na)^+^. Found: 256.0190. 

*2-[(5-(tert-Butoxycarbonyl)-2-thioxo-1,3,4-thiadiazol-3-yl)methyl]succinate* (**16**). 5-tert-Butylcarbamate-3H-1,3,4-thiadiazole-2-thione **(15)** (2.33 g, 10 mmol) was dissolved in 30 mL DMF, followed by the addition of 1.8 g (12 mmol) DBU and 1.9 g (12 mmol) dimethyl itaconate. The mixture was stirred for 40 hours under room temperature. Then, the solvent DMF was evaporated under high vacuum. The residue was loaded onto a silica gel column for purification. After column chromatography, 3.82 g white solid was collected, yield 81%, mp 140–142°C. ^1^H NMR (CDCl_3_): 7.89 ppm (s, NH), 4.42–4.62 ppm (m, CH_2_), 3.71 ppm (s, CH_3_), 3.66 ppm (s, CH_3_), 3.43–3.50 ppm (m, CH), 2.56–2.78 ppm (m, CH_2_), 1.49 ppm (s, 3CH_3_). ^13^C NMR (CDCl_3_): 183.33 ppm, 172.26 ppm (2 C=O), 171.60 ppm, 151.00 ppm, 84.50 ppm, 52.57 ppm, 52.04 ppm, 50.17 ppm, 39.97 ppm, 32.82 ppm, 27.98 ppm. IR: 3238 (m, N–H), 2980 (m, C–H), 1708 (s, C=O), 1587 (s, C=N), 1480 (s, NH bending), 1447 (s, C–H bending), 1317 (s, C–N), 1151 (s, C–O–C) cm^−1^. HRMS: Anal. Calcd. for C_14_H_21_N_3_O_6_S_2_: 414.0769 (M + Na)^+^. Found: 414.0778.

*5-tert-Butoxycarbonyl-3-[4 *′*-hydroxy-2 *′*-(hydroxymethyl) butyl]-1,3,4-thiadiazole-2-thione* (**17**). NaBH_4_ (0.08 g, 2.11 mmol) was stired with CaCl_2_ (0.12 g, 1.08 mmol) in 25 mL anhydrous THF for one hour. After the addition of 0.11 g (0.36 mmol) 2-[(5-(tert-butoxycarbonyl)-2-thioxo-1,3,4-thiadiazol-3-yl)methyl]succinate **(16)**, the mixture was stirred under nitrogen for 24 hours. 0.5 mL of methanol was added to quench the reaction. Then, the solid in the mixture was eliminated by filtration. The solvent in the filtrate was evaporated under reduced pressure. The residue was loaded onto a silica gel column for separation. 0.12 g clear gel-like product was collected via column chromatography, yield 96%. ^1^H NMR (CD_3_OD): 4.42–4.62 ppm (d, CH_2_), 3.62–3.65 ppm (t, CH_2_), 3.49–3.59 ppm (m, CH_2_), 2.28–2.38 ppm (m, CH), 1.55–1.68 ppm (m, CH_2_), 1.51 ppm (s, 3CH_3_). ^13^C NMR (CD_3_OD): 183.42 ppm, 154.88 ppm, 154.01 ppm, 83.87 ppm, 63.05 ppm, 60.66 ppm, 52.70 ppm, 38.83 ppm, 32.79 ppm, 28.33 ppm. IR: 3337 (m, N–H), 2926 (m, C–H), 1715 (s, C=O), 1578 (s, C=N), 1447 (m, NH bending), 1370 (s, C–N), 1150 (s, C–O) cm^−1^. HRMS: Anal. Calcd. for C_12_H_21_N_3_O_4_S_2_: 358.0865 (M + Na)^+^. Found: 358.0880. 

*5-Amino-3-(4 *′*-hydroxy-2 *′*-hydroxymethyl-butyl)-1,3,4-thiadiazole-2-thione* (**4**). 3 mL chilled 60% TFA in CH_2_Cl_2_ was added to 0.18 g (0.5 mmol) 5-tert-butoxycarbonyl-3-[4′-hydroxy-2′-(hydroxymethyl)butyl]-1,3,4- thiadiazole-2-thione **(17)** in an ice bath. After stirring at room temperature for 3 hours, the reaction mixture was extracted with 2 mL H_2_O twice. The saturated NaHCO_3_ solution was added to the aqueous phase to neutralize the acid. The solvent in the aqueous phase was evaporated under high vacuum, and then, the residue was loaded onto a column for purification. After column chromatography, 0.092 g clear liquid was collected, yield 78%. ^1^H NMR (CD_3_OD): 4.15–4.17 ppm (dd, CH_2_), 3.63–3.66 ppm (t, CH_2_), 3.50–3.59 ppm (m, CH_2_), 2.26–2.36 ppm (m, CH), 1.55–1.71 ppm (m, CH_2_). ^13^C NMR (CD_3_OD): 183.42 ppm, 154.01 ppm, 63.05 ppm, 60.66 ppm, 52.70 ppm, 38.83 ppm, 32.79 ppm. IR: 3321 (m, NH_2_), 3145 (m, NH), 2844 (m, C–H), 1616 (s, C=N), 1514 (m, NH bending), 1386 (m, C–N), 1097 (s, C–O) cm^−1^. HRMS: Anal. Calcd. for C_7_H_13_N_3_O_2_S_2_: 258.0341 (M + Na)^+^. Found: 258.0349. 

*5-Amino-3-(2 *′*,3 *′*-dihydroxypropyl)-1,3,4-thiadiazole-2-thione* (**7**). 5-tert-butylcarbamate-3H-1,3,4-thiadiazole-2- thione (**15**) (0.133 g, 1 mmol) was suspended in 20 mL anhydrous dioxane, followed by an addition of 0.028 g NaH. The mixture was stirred for one hour at room temperature under argon. Then, 0.10 g (1 mmol) of 3-chloro-1,2-propanediol was added dropwise. The mixture was refluxed for 24 hours under argon. After refluxing, the solvent was evaporated under reduced pressure. The residue was dissolved in 10 mL methanol, and then, the solid was filtered out. The solvent in the filtrate was evaporated to obtain the crude product of 5-tert-butoxycarbonyl-3-(2′,3′-dihydroxypropyl)-1,3,4- thiadiazole-2-thione **(18)**. Next, this crude product was stirred with 20 mL chilled 60% TFA in CH_2_Cl_2_ at room temperature for 3 hours. Then, this mixture was extracted with water three times and all the aqueous layers were combined. The saturated NaHCO_3_ solution was added to the aqueous phase to neutralize the acid. The solvent in the aqueous phase was evaporated under reduced pressure, and then, the residue was loaded onto a column for purification. After column chromatography, 0.14 g white solid was collected, yield 69%, mp 139–142°C. ^1^H NMR (CD_3_OD): 3.92–3.98 ppm (m, CH), 3.69–3.82 ppm (m, CH_2_), 3.49–3.57 ppm (m, CH_2_). ^13^C NMR (CD_3_OD): 169.90 ppm, 153.78 ppm, 71.07 ppm, 65.02 ppm, 50.13 ppm. IR: 3255 (m, NH_2_), 2900 (m, C–H), 1616 (s, C=N), 1514 (m, NH bending), 1386 (m, C–N), 1097 (s, C–O) cm^−1^. HRMS: Anal. Calcd. for C_5_H_9_N_3_O_2_S_2_: 208.0209 (M + H)^+^. Found: 208.0202. 

*(S)-5-Amino-3-(2 *′*,3 *′*-dihydroxypropyl)-1,3,4-thiadiazole-2-thione* (**5**). The preparation of **5 **followed the procedure for the synthesis of racemic mixture **7**, except that (R)-3-chloro-1,2-propanediol was used instead of the racemic 3-chloro-1,2-propanediol in the reaction. The melting point, IR, NMR, and MS results are identical with its enantiomer **6** and racemate **7**, [*α*]_D20_ = −33.0° (*c* = 1.0, CH_3_OH).

*(R)-5-Amino-3-(2 *′*,3 *′*-dihydroxypropyl)-1,3,4-thiadiazole-2-thione* (**6**). The preparation of **6 **followed the procedure for the synthesis of racemic mixture **7**, except that (S)-3-chloro-1,2-propanediol was used instead of racemic 3-chloro-1,2-propanediol in the reaction. The melting point, IR, NMR, and MS results are identical with its enantiomer **5** and racemate **7**, [*α*]_D20_ = +31.1° (*c* = 0.9, CH_3_OH).

*Bis-(5-Amino-1,3,4-thiadiazol-2-yl) Disulfide* (**19**) [43]. (a) 5-Amino-3H-1,3,4-thiadiazole-2-thione (**14**) (0.133 g, 1 mmol) was added to a solution of 1.5 mL H_2_O and 0.3 mL of glacial acetic acid. This mixture was cooled in an ice bath for 10 minutes. Then, a solution of 0.09 g (1 mmol) NaNO_2_ in 0.2 mL of H_2_O was added dropwise. After the mixture was stirred in an ice bath for 10 minutes, the dark yellow solid was filtered off and rinsed with two portions of 0.5 mL ethanol. After drying under reduced pressure, 0.098 g yellow solid was collected, yield 73%. (b) 5-Amino-3H-1,3,4- thiadiazole-2-thione **(14)** (0.133 g, 1 mmol) was added into a solution of 3 mL 50/50 ethanol/H_2_O mixture and kept in an ice bath. After the addition of 0.08 g NaNO_2_ to the mixture, 0.64 g melted SnCl_4_ ·5H_2_O was added dropwise. Immediately after the addition of SnCl_4_, a dark yellow solid formed. After stirring at room temperature for almost 20 minutes, the dark yellow solid was filtered off and rinsed twice with 0.5 mL cold water. After drying under reduced pressure, 0.091 g, the yellow solid was collected, yield 68%; mp 235-236°C (lit. mp 235–238°C [40]). ^1^H NMR (DMSO-d_6_): 7.76 (s, NH_2_) ppm. ^13^C NMR (DMSO-d_6_): 149.13 ppm, 172.68 ppm. IR: 3266 (m, anti-sym. NH_2_), 3069 (m, sym. NH_2_), 1633 (s, NH_2_ bending), 1494 (s, C=N), 1381 (m, C–N), 1135 (s, C=S) cm^−1^. MS *m/z*: 264.9 M^+^. HRMS: Anal. Calcd. for C_4_H_4_N_6_S_4_: 264.9453 (M + H)^+^. Found: 264.9459. 

Anal. Calcd. for C_4_H_4_N_6_S_4_: C, 18.17; H, 1.53; N, 31.79; S, 48.51. Found: C, 18.31; H, 1.44; N, 31.86; S, 48.10. 

## Figures and Tables

**Scheme 5:**